# Multidimensional Assessment of Orthorexia Nervosa: A Case-Control Study Comparing Eating Behavior, Adherence to the Mediterranean Diet, Body Mass Index, Psychological Symptoms, and Autonomic Arousal

**DOI:** 10.3390/nu17020317

**Published:** 2025-01-16

**Authors:** Sara Guidotti, Alice Fiduccia, Rosanna Sanseverino, Carlo Pruneti

**Affiliations:** Clinical Psychology, Clinical Psychophysiology and Clinical Neuropsychology Laboratory, Department of Medicine and Surgery, University of Parma, 43126 Parma, Italy; alice.fiduccia@unipr.it (A.F.); rosanna.sanseverino@studenti.unipr.it (R.S.); carlo.pruneti@unipr.it (C.P.)

**Keywords:** orthorexia nervosa, eating behavior, obsessive–compulsive symptoms, psychophysical stress, autonomic imbalance, autonomic nervous system, university students, mental health, somatization, Mediterranean diet

## Abstract

**Background:** The research on orthorexia nervosa (ON) has thoroughly outlined the connection between it and various mental disorders, including obsessive-compulsive disorders and eating disorders, in addition to stress. However, research has not considered psychophysical stress and other measures of psychophysical health, such as adherence to the Mediterranean diet. **Methods:** This cross-sectional and case-control research involved 63 students from the University of Parma, aged between 18 and 49 years. The ORTO-15 questionnaire was utilized to categorize the entire sample into two groups: one without orthorexia (score > 35) and another with orthorexia (score < 35). All subjects were assessed with the Psychophysiological Stress Profile (PSP) and completed the Eating Disorder Inventory-3 (EDI-3) and the Symptom Checklist-90-Revised (SCL-90-R). In addition, they were interviewed using the PREDIMED questionnaire to assess adherence to the Mediterranean Diet, and their body mass index (BMI) was calculated. **Results:** Subjects with orthorexia represented 38.10% of the total sample and reported a higher BMI than controls, although the PREDIMED score did not show a difference in adherence to the Mediterranean diet. The EDI-3 highlighted emotional dysregulation and hypercontrol in students with orthorexia, and a dissociation between subjective and objective measures of stress emerged. Particularly, the psychophysiological parameters of skin conductance, heart rate, and heart rate variability showed greater reactivity to stressful stimuli, but no difference was noted in psychological symptoms. **Conclusions:** These findings confirmed the presence of alterations in eating behavior in people with orthorexia as well as a higher BMI. It was hypothesized that hypercontrol might favor the perception of psychological well-being at a subjective level, although inadequate management of stress emerged at an objective psychophysiological level. Further studies are needed to highlight the causality between ON, hypercontrol, diet, and psychophysical stress, given that students with orthorexia present a dysregulation of emotions associated with greater autonomic arousal.

## 1. Introduction

In Western societies, thinness and beauty are often considered synonymous with health. The Guidelines for a Healthy Diet [1] introduce weight control in the first chapter. The focus is on physical activity, with suggestions to conduct unstructured physical activity when possible, such as climbing stairs and walking. By way of illustration, the first point states: “Your weight depends mainly on you”, making the overweight adult population responsible for their pathology, asking them to reduce their caloric intake and increase physical activity. However, weight gain results from a complex interaction between many factors, characterized largely by lifestyle (i.e., diet, sleep, and physical activity) and sociocultural and psychological factors. Specifically, an increased caloric intake is influenced by stress, anxiety, depression, and eating disorders.

Yau and colleagues [2] analyzed the relationship between stress and eating behavior, calculating that about 40% of people reduce their caloric intake, about 40% of people increase it, and about 20% do not change their eating behavior during periods of stress. The authors hypothesized that this may depend on the type of stress. Severe stress could lead to hypophagia, while mild stress could lead to hyperphagia. In this regard, those who increase their caloric intake usually consume hyper-palatable foods rich in salt or sugar, as well as the frequency of snacks. Among the coping mechanisms, emotional eating deserves attention, that is, the intake of food in response to strong negative emotions such as boredom, anger, sadness, loneliness, and stress. People who adopt this coping strategy tend to seek highly palatable foods capable of activating the reward system based on dopamine release. This neurotransmitter is involved in addiction processes, which is why some researchers hypothesized the existence of food addiction. People who use food to improve their mood tend to consume large amounts of simple carbohydrates, progressively developing a real dependence on them, known as carbohydrate cravings [3]. During episodes of emotional eating, subjects do not perceive a real sensation of physical hunger and cannot feel a sense of satiety. A vicious circle is often established because excessive amounts of food are consumed to relieve frustration, with a temporary improvement in mood [4]. On the other hand, the weight gain resulting from these episodes leads to a further sense of frustration, fueling the cycle [5,6]. This way of managing emotions can contribute to the development of eating disorders such as Binge Eating Disorder (BED) and Bulimia Nervosa (BN), both characterized by the presence of binges, and can favor the onset of obesity.

Another relevant coping mechanism is self-control. People who adopt this strategy try to repress their emotions and sensations, including hunger. This behavior is typical of Anorexia Nervosa (AN) [7]. Caloric intake is also tightly controlled in Orthorexia Nervosa (ON), in which the focus is on checking the quality of food and nutrition, in general. ON and AN share several characteristics, besides hypercontrol, such as perfectionism and concern for physical appearance. In some cases, ON can serve as a coping mechanism for anorexic patients, shifting the focus from extreme caloric restriction to the selection of healthy foods, resulting in the alleviation of eating disorder symptoms [8]. ON is an emerging disorder that meets the diagnostic criteria of both Nutrition and Eating Disorders (NED) and Obsessive–Compulsive Disorder (OCD). For this reason, the scientific community is still debating the categorization of orthorexia.

To this purpose, Donini and colleagues [9] wrote a consensus document in which 93% of the scholars agreed to include orthorexia among NED, but it was also corroborated that the disorder presents characteristics of OCD. Specifically, orthorexic subjects have intrusive thoughts about food; they also tend to avoid unhealthy foods that can damage health; and they are rigidly adherent to preparation methods. They may also engage in ritualized behaviors, such as meticulously weighing food. It is essential to remember that orthorexic subjects do not feel bothered by these obsessions and compulsions concerning food and health, which is why their behavior is said to be ego-syntonic or in line with their self-image. On the other hand, patients with OCD, who present obsessions and compulsions that cause discomfort (ego-dystonic), can still develop a comorbid ON, especially because the two disorders share traits of rigidity and perfectionism.

In ON, weight loss may not always be desired, but nutritional deficiencies may be found as well as hormonal imbalances. Donini and colleagues [9], again, state that vegan and vegetarian eating habits can be considered risk factors for the development of ON, but only in the case in which the elimination of foods of animal origin is due to a health motivation and not an ethical one. On the contrary, if the exclusion of some foods from the diet is attributable to clinical dietary prescriptions, economic conditions, or religious and ethical motivations, this type of diet cannot be considered a risk factor for ON. Avoidance of specific food groups or certain cooking methods for fear of health repercussions, typical of ON, can also be a diagnostic criterion for avoidant/restrictive food intake disorder (ARFID). However, patients with ARFID fear the immediate consequences of eating a specific food (e.g., choking), while patients with ON fear the long-term effects (e.g., cardiovascular disease).

Nonetheless, if the subject does not experience stress in following their dietary rules, orthorexia may be considered healthy orthorexia, which could favor an improvement in their health status. The motivations behind healthy food choices may be different in subjects with ON and healthy orthorexia. In the first case, the main motivation would be weight control, while in the second case, it would be the healthy aspect of food [10].

Furthermore, Strahler and colleagues [11] found that subjects with ON, despite following a healthy lifestyle, had lower life satisfaction and higher levels of stress. Notwithstanding, it is not possible to identify whether subjects with ON tend to become more stressed than control subjects or whether stressed subjects tend to control their lifestyle as a coping mechanism to stress.

In light of the previous research, this study aimed to examine the psychophysical stress and psychological symptoms of individuals with ON. Additionally, eating behavior and adherence to the Mediterranean diet as well as body mass index (BMI) were also compared between the two groups. The hypotheses that guided this research were: (1) people with ON have greater adherence to the Mediterranean diet; (2) people with ON have eating behavior dysfunctions and increased BMI compared to controls; (3) people with ON have higher levels of psychophysiological activation; (4) people with ON have higher levels of psychological symptoms (i.e., anxiety, depression, and obsessions and compulsions).

## 2. Materials and Methods

### 2.1. Participants

The study design was an exploratory case-control study. To validate the research hypotheses, 63 students (29 males and 34 females) were consecutively recruited. Their age ranged from 18 to 49 years. Inclusion criteria included (1) age > 18 years, (2) completion of informed consent, (3) absence of psychiatric and/or neurological syndromes (i.e., previous head trauma, epilepsy, etc.), and/or physical diseases (i.e., sensory disturbances of vision and/or hearing) that could hinder the administration of the questionnaires and the psychophysiological assessment procedure; and (4) no intake of drugs with evident autonomic effect in the last three months.

### 2.2. Procedure

An invitation to participate in the study was presented on some posters on the bulletin boards of the University of Parma as well as being distributed through the university mailing list. Participants went to the Clinical Psychology, Clinical Psychophysiology, and Clinical Neurophysiology Laboratories of the Department of Medicine and Surgery to obtain a description of the objectives of the study and to express their consent to participate. A clinical psychologist regularly registered on the national list was also available at the end of the experimental procedure for the debriefing as well as to return the individual results within an interview covered by professional confidentiality.

The experimental procedures conducted complied with the 1964 Helsinki Declaration of the World Medical Association as well as the 2005 Universal Declaration on Bioethics and Human Rights of UNESCO.

### 2.3. Measures

Orthorexia was measured through the italian version of the Orthorexia Nervosa Questionnaire-15 (ORTO-15) [12], which consists of 15 items with 4-point Likert scale answers (always, often, sometimes, never). Scores are computed by summing the responses to each item, with a value of 1 indicating orthorexic traits and a value of 4 reflecting normal eating behaviors. The total score can range from 15 to 60, with a cut-off value set at <35. Scores lower than 35 indicate the presence of ON, whereas scores higher than 35 exclude the presence of ON. The Cronbach’s Alpha of the study was equal to 0.72.

The PREDIMED questionnaire (the acronym derived from the Spanish study “PREvención con DIeta MEDiterránea”) was used to measure adherence to the Mediterranean Diet [13], which is composed of 14 items investigating the intake and quantity of extra virgin olive oil and the frequency of intake of fruit, vegetables, nuts, legumes, red meat, poultry, fish, animal fats, sugary drinks, sweets, and fried foods [14]. Each item can receive a score of 0 or 1. The sum of the scores corresponds to the PREDIMED score, indicating low (from 0 to 5), medium (from 6 to 9), or high (greater than or equal to 10) adherence to the Mediterranean diet [15].

At the end of the interview, each participant was asked to step on a mechanical scale with an altimeter to simultaneously determine their weight and height. Once the weight and height parameters were obtained, the body mass index (BMI) was calculated, defined as body mass (expressed in kilograms) divided by the square of the height (expressed in meters). The BMI, expressed in units of kg/m^2^, is an indicator of healthy weight status as well as one of the indirect indices of eating habits and lifestyle when there are no organic causes of underweight or overweight [16].

The Eating Disorder Inventory-3 [17] is a self-assessment questionnaire useful for investigating alterations in eating behavior. There are six composite scores resulting from the sum of twelve sub-scales. Specifically, Risk of eating disorders (EDRC), Ineffectiveness (IC), Interpersonal problems (IPC), Affective problems (APC), Hypercontrol (OC), and Global psychological maladjustment (GPMC) are the composite scores. Drive for thinness, Bulimia, Body dissatisfaction, Low self-esteem, Personal alienation, Interpersonal insecurity, Interpersonal alienation, Interoceptive deficits, Emotional dysregulation, Perfectionism, Asceticism, and Fear of maturity are the sub-scales. Among the various scales, Cronbach’s Alpha ranges from a value of 0.70 to a value of 0.94.

The Symptom Checklist-90-Revised (SCL-90-R) [18,19] was used to measure psychological symptoms present in the week preceding the completion of the standardized questionnaire. The 90 total items comprise the following different clinical scales, which are Somatization, Obsessions and Compulsions, Interpersonal Sensitivity, Depression, Anxiety, Hostility, Phobic Anxiety, Paranoid Ideation, and Psychoticism. A five-point Likert scale (from 0, which corresponds to “not at all”, to 4, which corresponds to “very often”) is used to answer the items. The Global Severity Index (GSI) corresponds to the mean score of all of the items; therefore, it is an overall indicator of the current level or intensity of the symptomatology complained of by the person involved. The authors ensured that a score equal to or greater than 63 in two or more clinical scales or the GSI scale suggests the presence of a clinically significant state of distress. Cronbach’s Alpha between the various clinical scales ranges between 0.67 and 0.87.

Once it was ensured that the subjects had not consumed coffee, alcohol, or nicotine in the two hours preceding the meeting, a Psychophysiological Stress Profile (PSP) [20] was recorded using Biograph Procomp Infiniti 5.0 software (Thought Technology Ltd., Montreal, QC, Canada). The PSP procedure begins by asking the subjects to keep their feet flat on the floor (at 45 degrees) and their arms stretched out along the armrests of the chair in which they are comfortably seated. The room temperature is also controlled (19–21 °C). There are seven recording phases: (1) Baseline, the subject is asked to close their eyes and relax; (2) Stressor 1, the subject is subjected to the Stroop test (computerized version); (3) Rest 1, the subject is asked to relax as much as possible; (4) Stressor 2, the subject is asked to solve a serial subtraction task (for example, consecutively subtract the number 7 starting from 1008); (5) Rest 2, as in Rest 1; (6) Stressor 3, the person is asked to summarize a significant life event; and (7) Rest 3, as in Rest 1. The psychophysiological parameters measured were SC, HR, and HRV. SC is detected by passing a very low-intensity electric current between two 1 cm^2^ silver circular electrodes applied to the index and middle fingers of the non-dominant hand. The raw value is converted to a percentage change (SC%). HR was measured by blood volume pressure (BVP) detection performed with a blood volume pulse sensor (also known as a photoplethysmograph, PPG) that was applied to the middle finger of the dominant hand. The PPG, by sending infrared light to the skin, measures the amount of light absorbed and, therefore, the changes in blood flow [21]. The frequency domain of HRV analysis was analyzed by extrapolating the values of the low-frequency (LF; 0.04–0.15 Hz) and high-frequency (HF; 0.15–0.40 Hz) bands. According to Kuo et al. [22], the same values were considered according to the power spectrum analysis in which the LF and HF values are converted into normalized units (0–100) expressing the percentage of the total variance (LF% and HF%, respectively). For the parameters of SC%, LF%, and HF%, the values of baseline, reactivity, and recovery were considered. Reactivity and recovery were obtained according to the procedure of Laborde et al. [23]. Reactivity is the difference between the stressor phases and the baseline phase (i.e., Stress 1—Baseline = Reactivity 1; Stress 2—Baseline = Reactivity 2; and Stress 3—Baseline = Reactivity 3) while recovery is calculated by subtracting the stressor phase values from the rest phases (i.e., Rest 1—Stress 1 = Recovery 1; Rest 2—Stress 2 = Recovery 2; and Rest 3—Stress 3 = Recovery 3).

### 2.4. Statistical Analysis

SPSS was used to conduct all of the statistical analyses planned (Version 28.0.1.0; IBM Corp., Armonk, NY, USA). Initially, descriptive statistics were calculated, including the mean (M) and standard deviation (SD) of variables. Because all the assumptions necessary for conducting parametric statistics were satisfied, differences between groups regarding socio-demographic characteristics (such as age, gender, educational level, marital status, and education level) were assessed using the Chi-square Test and Independent Samples’ *t*-test.

Consequently, differences in adherence to the Mediterranean Diet (PREDIMED score), BMI, eating behavior (EDI-3), psychological symptoms (SCL-90-R), and psychophysiological values (SC%, HR, LF%, and HF%) were assessed. The significance level was set at 0.05.

## 3. Results

Initially, the cut-off score of 35 on the ORTO-15 was used to identify a group of university students with ON. Among these participants, those scoring below 35 numbered 24, representing 38.10% of the total sample. When examining the socio-demographic factors, the group of students with ON did not differ from the controls in any of the observed variables, except for the BMI measurement (Table 1).

Looking at eating behavior (Table 2), a slight tendency to desire thinness emerged, although it was only at the limits of statistical significance. Statistically significant evidence concerns the clinical and composite scales. Specifically, relational and affective problems, as well as hypercontrol, seemed to characterize the group of orthorexic subjects. Consistently, the clinical scales showed interpersonal insecurity and emotional dysregulation associated with hypercontrol and asceticism.

However, no significant differences emerged between the groups when looking at psychological symptoms (Table 3). The difference in the somatization scale of the SCL-90-R was at the limits of the statistical threshold, indicating mild somatic manifestations of stress and anxious activation.

Regarding psychophysical stress, significant differences emerged concerning the HRV LF and HF baseline values, indicating a sympathovagal imbalance at rest (Table 4).

The greater sympathetic activity was recorded by observing the skin conductance reactivity 1 and 2 and recovery 2 values. Greater cardiac reactivity was observed in reactivity 3 and recovery 3, as demonstrated by the higher heart rate in people with orthorexia. Greater sympathovagal oscillations in response to stress were attested by the LF reactivity 1, 2, and 3 values. In parallel, a significantly higher vagal tone in the orthorexic group was observed in response to all three stressful stimuli. Nonetheless, the recovery 3 value of the same group was also greater than that of the controls (Table 5).

## 4. Discussion

The research hypotheses that guided the present study aimed to shed light on some clinical-psychological and psychophysiological variables involved in ON. Nevertheless, the link between ON and some socio-demographic measures and anthropometric and diet-related ones was explored.

First, the comparison between orthorexic and control subjects verified no significant difference in gender or age, being in line with various previous studies [24,25,26,27] but the opposite of the author who previously found correlations with gender and age [28,29].

Even looking at the level of education, no differences were appreciated, although the literature reports a recurrent imbalance in favor of a lower level of education connected to ON, as described by Donini et al. [30], Depa et al. [31], and Malborg et al. [32]. Interestingly, the study of Bagci Bosi et al. [33] verified a higher prevalence of ON among doctors. Even though our results cannot be read entirely in light of this evidence, it should be noted that most of the students in the present study were enrolled in a course for healthcare professionals. Greater attention to nutrition and a healthy lifestyle could explain the high prevalence of ON in our sample.

Nonetheless, the PREDIMED score did not differ between the two groups. In other words, students with ON had similar adherence to the Mediterranean diet as controls. This finding is discordant with the increased BMI. Specifically, our study confirms previous studies that found a higher BMI among people with ON. Both Bundros et al. [34] and Oberle et al. [35] reported a correlation between increased BMI and ON. Nevertheless, the BMI of 24.8 of Bundros et al. [34] is similar to that of our sample of orthorexic students, who are borderline overweight. Still reflecting information already present in the literature, the reason for this result is not yet clear. It could be hypothesized that some of them were returning from a low-calorie diet or that there were alterations in eating behavior that facilitated an increase in BMI.

Among the interesting results of this research, there are similarities with EDs. It seems that individuals with ON reported greater emotional dysregulation, which might represent a risk factor for both the development of stress-related symptoms and autonomic imbalance [36], as well as emotional eating in response to stress [37]. Notwithstanding, more adequate tools exist for assessing emotional eating since emotional dysregulation, as measured by the EDI-3, may be associated with an increased risk of binge eating, eating without hunger, and obesity risk [38]. Emotional eating, when driven by emotional dysregulation, can represent a factor able to perpetuate a chronic response to stress, being linked to feelings of guilt [5,6].

These dynamics may be associated with hypercontrol, as individuals with ON have been shown to have internalized rules based on obsessive tendencies [39] that they share with other EDs, such as AN [9]. Among the most relevant aspects of our research is the composite score of hypercontrol, which was significantly higher in students with ON compared to controls. Partially discordant with current literature is the absence of relevant differences in the OCD scale of the SCL-90-R. In this regard, it should be noted that the score was high in both groups (>63 T points). Therefore, students with ON could still have obsessive thoughts that they try to regulate by engaging in actions aimed at neutralizing them [40]. To illustrate, ON could be considered as compulsive as EDs’ behavior. The tendency to engage in compulsive behaviors despite their negative consequences is assumed to be the hallmark of OCD by researchers, which is conceptualized as a disorder of self-control and behavioral inhibition [41,42]. Examples include neuroscientific research indicating a significant impairment in cognitive flexibility among individuals with obsessive-compulsive symptoms [43,44], likely due to under-activation of prefrontal regions [45,46] and limbic structures involved in the emotional processing of stimuli [46,47].

Additionally, it could be relevant to note a greater tendency to somatic manifestations of stress and negative affectivity. However, no significant differences on the somatization scale of the SCL-90-R were found. Similarly, different from what was initially hypothesized, no psychological symptoms on the SCL-90-R were significantly higher in students with ON compared to controls. These data might suggest the presence of a dissociation between subjective and objective aspects, as if behavioral compulsions allowed the maintenance of the perception of well-being. Consistently, it could be hypothesized that it would be only the transgressions from the strict diet that instill exaggerated fears and negative physical sensations [48].

The hypotheses that guided the comparison between psychophysiological parameters were validated, as significant differences emerged in the values of reactivity and recovery of skin conductance, heart rate, and heart rate variability when dividing the total sample into subjects with ON and controls. In particular, it was observed that subjects with ON would show an alteration of their basal levels, a greater reactivity to stress, and a lower capacity to restore basal levels. These results may suggest that individuals with ON show autonomic hyperarousal. Specifically, the results could suggest a predominance of the sympathetic activity of the autonomic nervous system. In light of the findings of our previous research that found significantly higher levels of motor and behavioral hyperactivity [39], it could be hypothesized that the hyperactive lifestyle observed in subjects with ON could contribute to autonomic hyperarousal as well as to the production of somatic symptoms. Nevertheless, the data of HRV-HF, a parameter that indicates vagal tone, are particularly relevant. Our results seem to be in line with those obtained by other researchers on clinical populations. Specifically, an increase in HF has been observed in response to stress in patients with depressive syndrome [49], suggesting that a compensatory mechanism of the parasympathetic system toward the sympathetic one may exist.

The psychophysiology of ON has never been explored in previous studies [39], and it represents an innovative aspect of our research. In light of these assumptions, the obsessive component of ON could be seen as a form of hypercompensation associated with emotional dysregulation and vulnerability to stress [50]. This aspect could explain the autonomic imbalance and higher BMI as possible outcomes of the resulting tendency toward emotional eating. Contrary to what was expected, our study did not find differences in psychological symptoms. Future directions could focus on investigating their defense mechanisms and coping styles more specifically.

While our research offers important insights that might have clinical relevance, it is not without limitations. Firstly, the study’s cross-sectional design prevents us from establishing causal relationships among the variables examined. For instance, it was hypothesized that emotional dysregulation may facilitate the stress vulnerability and the consequent emotional eating of people with ON, in which obsessive thoughts could be dysregulation as a strategy to control negative emotions. In contrast, stress may intensify obsessive thoughts and contribute to emotional dysregulation. Furthermore, the ad hoc sampling method may not accurately represent the university student population, as participants might have been driven by curiosity or a desire to gain insight into specific aspects of themselves. Nonetheless, it is crucial to thoughtfully consider the reported prevalence of ON (38%), even if it aligns with the existing literature on university students, which ranges from 7 to 83% depending on the instruments and cut-offs used [51]. Additionally, the relationships among ON, emotional dysregulation, hypercontrol, autonomic arousal, BMI, dietary patterns, and psychological symptoms warrant further investigation using more precise measurement techniques. As already mentioned previously, these findings could be associated with the analysis of defense mechanisms and coping strategies.

Despite existing limitations, our study seems to highlight the particular interaction between ON and various psychological and psychophysiological aspects. Nonetheless, the high prevalence of participants exhibiting ON aligns with findings related to anxiety, depression, and eating disorders, as noted in research examining psychopathological disorders among university students [52,53]. Alarmingly, the elevated levels of emotional and behavioral disorders underscore the necessity for enhanced emotional regulation support for this social group, along with the implementation of functional and adaptive coping strategies for managing stress and negative emotions.

Recognizing that ON significantly impacts various aspects of an individual’s overall functioning, a diverse range of healthcare professionals needs to collaborate effectively. This team may comprise nutritionists and psychologists, each playing an important role in fostering body-mind integration. Psychologists bear a critical responsibility in advocating for the incorporation of effective stress management techniques within therapeutic programs to prevent the adoption of maladaptive eating behaviors as a means to cope with emotions. By doing so, they can empower individuals to cultivate healthier coping strategies and enhance their resilience in the face of life’s challenges. In this context, autonomic hyper-arousal underscores the presence of inadequate emotional self-regulation skills coupled with heightened vulnerability to stress. Tice and colleagues [54] demonstrated that emotional eating, accompanied by subsequent hypercontrol, could serve as a manifestation of poor impulse control. The authors observed that individuals exposed to emotionally stressful situations often struggle to pursue long-term goals, opting for immediate rewards, such as binge eating or the consumption of alcohol and other psychoactive substances, to manage negative emotions.

Individuals susceptible to stress with somatic consequences (such as autonomic hyperarousal and somatization) may place an overwhelming burden on mental health departments and, more broadly, on the services provided by the national health system due to their physical manifestations. This phenomenon often leads to the so-called “medical shopping”, particularly in cases of stress-related physical syndromes, where psychological factors are insufficiently explored and functional symptoms unaddressed. Furthermore, individuals with ON may seek guidance from nutritionists and dieticians to adhere to diets that align with their obsessive standards. Conversely, national policies could focus on preventing these types of mental disorders. On the one hand, stress management should be prioritized in public health, with primary and secondary prevention strategies designed to enhance understanding of emotions, stress, and anxiety while also promoting the identification of risk factors associated with stress-related symptoms. On the other hand, preventing eating disorders is crucial and should involve large-scale interventions targeted primarily at students of all ages. It would be beneficial for institutions to engage nutritionists in providing guidance not only on general dietary recommendations but also on appropriate portion sizes and the normalization of the physiological enjoyment and satisfaction derived from eating. By promoting this balanced perspective, nutritionists can indirectly support an individual’s holistic well-being, ensuring that health is pursued in a manner that is both healthy and sustainable.

## 5. Conclusions

Our study aimed to analyze some clinical-psychological and psychophysiological variables that can distinguish people with ON. The analyses manifested a dissociation between objective and subjective measures. Specifically, the eating behavior of people with ON seems to be characterized by emotional alterations associated with hypercontrol. Consistently, a greater sympathetic activity at rest and in response to emotional stimuli was certified. However, a level of subjective distress in terms of greater psychological symptoms compared to controls did not emerge. It has been hypothesized that there may be a dissociation between the well-being experienced at a subjective level and the anxious activation at a psychophysiological level. People with orthorexia may use eating behavior as a strategy characterized by hypercontrol for regulating emotions. However, the repression of emotions would not favor the correct management of stress, which would be evident in a psychophysiological investigation.

## Figures and Tables

**Table 1:** Comparison of socio-demographic characteristics between the non-orthorexic and the orthorexic groups.

Variable	ORTO-15 Score > 35 (*n* = 39)	ORTO-15 Score < 35 (*n* = 24)	Total Sample (*n* = 63)	t or χ^2^	*p*
Age (years), M (SD)	24.38 (4.00)	26.91 (7.06)	25.32 (5.43)	t (62) = −1.81	0.08
Gender, *N* (%)				χ^2^ (1, *N* = 62) = 2.36	0.12
Male	15 (38.5%)	14 (58.3%)	29 (46%)		
Female	24 (61.5%)	10 (41.7%)	34 (54%)		
Occupation, *N* (%)				χ^2^ (1, *N* = 62) = 0.89	0.35
Student	33 (84.6%)	18 (75%)	51 (81%)		
Student + worker	6 (15.4%)	6 (25%)	12 (19%)		
Education Level, *N* (%)				χ^2^ (1, *N* = 62) = 2.74	0.10
High school graduation	16 (41%)	15 (62.5%)	31 (49.2%)		
University degree	23 (59%)	9 (37.5%)	32 (50.8%)		
Marital status, *N* (%)				χ^2^ (1, *N* = 62) = 1.21	0.27
Unmarried	36 (93.3%)	20 (83.3%)	56 (88.9%)		
Married/cohabitant	3 (7.7%)	4 (16.7%)	7 (11.1%)		
Children, *N* (%)				χ^2^ (1, *N* = 62) = 2.47	0.12
No	38 (97.4%)	21 (87.5%)	59 (93.7%)		
Yes	1 (2.6%)	3 (12.5%)	4 (6.3%)		
Clinical measures					
BMI, M (SD)	22.61 (3.12)	24.90 (4.77)	24.90 (4.77)	t (62) = −2.17	0.02
PREDIMED score, M (SD)	7.81 (3.12)	7.95 (1.47)	7.95 (1.47)	t (62) = −0.31	0.38

**Table 2:** Comparison of eating behavior between the non-orthorexic and the orthorexic groups.

Variable	ORTO-15 Score > 35 (*n* = 39)		ORTO-15 Score < 35 (*n* = 24)		t (62)	*p*	Cohen’s D
	M	SD	M	SD			
Risk Scales							
Drive for Thinness	32.97	28.05	39.13	29.92	−1.45	0.07	−0.21
Bulimia	35.55	31.23	38.21	30.80	−0.58	0.28	−0.09
Body Dissatisfaction	36.42	24.40	36.03	27.02	−0.10	0.46	0.02
Clinical Scales							
Low Self-Esteem	44.06	27.94	41.14	26.86	0.72	0.24	0.11
Personal Alienation	45.67	28.99	43.87	27.45	0.43	0.33	0.06
Interpersonal Insecurity	51.65	29.18	43.62	28.33	1.89	0.03	0.28
Interpersonal Alienation	47.63	27.36	42.94	27.94	1.16	0.13	0.17
Interoceptive Deficits	47.96	29.17	51.96	27.85	−0.95	0.17	−0.14
Emotional Dysregulation	39.73	26.67	47.83	30.71	−1.94	0.03	−0.29
Perfectionism	45.12	30.68	54.87	27.63	−2.25	0.01	−0.33
Asceticism	43.58	26.92	51.26	26.09	−1.96	0.03	−0.29
Maturity Fears	48.97	31.77	49.85	31.11	−0.19	0.43	−0.03
Composite scales							
Eating Disorder Risk	35.58	24.91	38.85	27.24	−0.86	0.20	−0.13
Ineffectiveness	45.99	27.58	43.72	25.93	0.57	0.28	0.08
Interpersonal Problems	51.50	27.91	44.29	27.45	1.76	0.04	0.26
Affective Problems	45.61	26.73	52.88	26.09	−1.87	0.03	−0.28
Overcontrol	45.01	27.33	55.45	26.00	−2.65	0.004	−0.39
Global Psychological Maladjustment	48.81	26.11	51.33	25.24	−0.67	0.25	−0.10

**Table 3:** Comparison of psychological symptoms between the non-orthorexic and the orthorexic groups.

Variable	ORTO-15 Score > 35 (*n* = 39)		ORTO-15 Score < 35 (*n* = 24)		t (62)	*p*	Cohen’s D
	M	SD	M	SD			
Somatization	56.65	6.60	59.37	7.72	−1.33	0.09	−0.19
Obsession–Compulsion	67.44	7.35	69.62	7.29	−0.92	0.18	−0.13
Interpersonal Sensitivity	63.90	6.53	64.05	6.19	−0.05	0.48	−0.01
Depression	63.86	9.45	65.12	9.68	−0.52	0.30	−0.08
Anxiety	61.59	6.27	63.95	7.09	−0.90	0.19	−0.13
Hostility	57.68	3.13	57.38	3.31	0.09	0.46	0.01
Phobic Anxiety	55.99	3.77	55.85	3.28	0.06	0.48	0.01
Paranoid Ideation	65.42	4.51	66.82	4.81	−0.55	0.29	−0.08
Psychoticism	66.24	5.29	69.88	6.30	−1.09	0.14	−0.16
Global Severity Index	55.16	1.27	54.84	1.27	−1.06	0.15	−0.15

**Table 4:** Comparison of psychophysiological values at baseline between the non-orthorexic and the orthorexic groups.

Variable	ORTO-15 Score > 35 (*n* = 39)		ORTO-15 Score < 35 (*n* = 24)		t (62)	*p*	Cohen’s D
	M	SD	M	SD			
Skin Conductance (%)	33.01	24.20	43.90	39.90	−1.33	0.10	−0.35
Heart Rate (bpm)	73.90	13.22	74.62	13.28	−0.27	0.42	−0.06
HRV-LF% (nu)	37.08	16.43	45.60	18.47	−1.87	0.03	−0.49
HRV-HF% (nu)	42.13	18.20	29.63	14.30	2.81	0.0030	0.74

**Table 5:** Comparison of psychophysiological values reactivity and recovery between the non-orthorexic and the orthorexic groups.

Variable	ORTO-15 Score > 35 (*n* = 39)		ORTO-15 Score < 35 (*n* = 24)		t (62)	*p*	Cohen’s D
	M	SD	M	SD			
Skin conductance (%)							
Reactivity 1	16.27	16.55	29.95	33.40	−2.14	0.02	−0.56
Reactivity 2	21.94	22.25	42.88	54.23	−2.11	0.02	−0.56
Reactivity 3	27.64	28.80	29.74	65.92	−0.17	0.43	−0.05
Recovery 1	5.34	9.09	11.25	20.16	−1.60	0.06	−0.42
Recovery 2	6.44	8.74	12.45	19.34	−1.66	0.05	−0.44
Recovery 3	5.60	7.44	5.12	21.48	0.13	0.45	0.03
Heart Rate (bpm)							
Reactivity 1	12.05	11.48	10.19	7.54	0.69	0.25	0.18
Reactivity 2	13.07	12.03	10.89	6.60	0.80	0.21	0.21
Reactivity 3	18.13	14.97	9.22	18.07	2.08	0.02	0.55
Recovery 1	12.91	9.00	9.93	7.02	1.36	0.09	0.34
Recovery 2	13.97	9.88	10.44	4.62	1.61	0.06	0.43
Recovery 3	16.71	11.08	10.14	8.01	2.47	0.0080	0.65
HRV-LF% (nu)							
Reactivity 1	7.97	20.07	0.98	17.11	1.39	0.09	0.34
Reactivity 2	8.98	20.85	−2.79	24.75	1.99	0.03	0.53
Reactivity 3	1.23	19.04	−11.76	23.83	2.35	0.01	0.62
Recovery 1	5.59	23.23	9.19	15.30	−0.66	0.26	−0.17
Recovery 2	4.82	22.72	0.73	23.67	0.67	0.25	0.18
Recovery 3	−1.80	18.71	−5.33	21.78	0.67	0.25	0.18
HRV-HF% (nu)							
Reactivity 1	−7.55	20.72	0.49	13.35	−1.66	0.05	−0.44
Reactivity 2	−10.31	21.62	2.71	20.69	−2.32	0.01	−0.61
Reactivity 3	−4.79	18.30	9.81	18.76	−2.99	0.0020	−0.79
Recovery 1	−5.96	19.63	−6.78	18.30	0.16	0.44	0.04
Recovery 2	−6.17	19.98	−1.05	22.34	−0.93	0.18	−0.25
Recovery 3	1.26	16.44	9.89	19.54	−1.85	0.04	−0.49

## Data Availability

The raw data supporting the conclusions of this article will be made available by the authors upon request.
